# Cognitive Reserve and Related Constructs: A Unified Framework Across Cognitive and Brain Dimensions of Aging

**DOI:** 10.3389/fnagi.2022.834765

**Published:** 2022-05-27

**Authors:** William S. Kremen, Jeremy A. Elman, Matthew S. Panizzon, Graham M. L. Eglit, Mark Sanderson-Cimino, McKenna E. Williams, Michael J. Lyons, Carol E. Franz

**Affiliations:** ^1^Department of Psychiatry, University of California, San Diego, La Jolla, CA, United States; ^2^Center for Behavior Genetics of Aging, University of California, San Diego, La Jolla, CA, United States; ^3^Center of Excellence for Stress and Mental Health, VA San Diego Healthcare System, San Diego, CA, United States; ^4^Department of Psychology, San Diego State University, San Diego, CA, United States; ^5^Department of Psychological & Brain Sciences, Boston University, Boston, MA, United States

**Keywords:** cognitive resilience, brain resilience, cognitive reserve maintenance, peak reserve, current reserve, reverse causation

## Abstract

Cognitive reserve and related constructs are valuable for aging-related research, but consistency and clarification of terms is needed as there is still no universally agreed upon nomenclature. We propose a new set of definitions for the concepts of reserve, maintenance, and resilience, and we invoke parallel concepts for each that are applicable to cognition and to brain. Our definitions of reserve and resilience correspond reasonably well to dictionary definitions of these terms. We demonstrate logical/methodological problems that arise from incongruence between commonly used conceptual and operational definitions. In our view, cognitive reserve should be defined conceptually as one’s total cognitive resources at a given point in time. IQ and education are examples of common operational definitions (often referred to as proxies) of cognitive reserve. Many researchers define cognitive reserve conceptually as a property that allows for performing better than expected cognitively in the face of aging or pathology. Performing better than expected is demonstrated statistically by interactions in which the moderator is typically IQ or education. The result is an irreconcilable situation in which cognitive reserve is both the moderator and the moderation effect itself. Our proposed nomenclature resolves this logical inconsistency by defining performing better than expected as cognitive resilience. Thus, in our usage, we would test the hypothesis that high cognitive reserve confers greater cognitive resilience. Operational definitions (so-called proxies) should not conflate factors that may influence reserve—such as occupational complexity or engagement in cognitive activities—with cognitive reserve itself. Because resources may be depleted with aging or pathology, one’s level of cognitive reserve may change over time and will be dependent on when assessment takes place. Therefore, in addition to cognitive reserve and cognitive resilience, we introduce maintenance of cognitive reserve as a parallel to brain maintenance. If, however, education is the measure of reserve in older adults, it precludes assessing change or maintenance of reserve. Finally, we discuss consideration of resistance as a subcategory of resilience, reverse causation, use of residual scores to assess performing better than expected given some adverse factor, and what constitutes high vs. low cognitive reserve across different studies.

## Introduction

The U.S. National Institute on Aging (NIA) sponsored the Reserve and Resilience Workshop Collaboratory led by Dr. Yaakov Stern^1^. The NIA’s request for this type of workshop underscores the importance of these concepts in research on aging. Stern’s work in this area has yielded very useful and highly influential constructs in aging and dementia research (Stern, 2012; Stern et al., 2020). A seminal finding that has spurred interest in cognitive reserve was Stern’s observation that adults with higher educational attainment tended to have later onset of dementia, but once they did reach a point of significant cognitive impairment they then had a more rapid decline in function than those with lower education (Stern et al., 1995, 1999). The simple, yet powerful, idea is that those with more education were able to withstand more brain pathology, but having more pathology once impairment becomes manifest, they in turn may undergo a more rapid subsequent decline.

Several recent reviews and commentaries on reserve and related issues have been published (Arenaza-Urquijo and Vemuri, 2018, 2020; Cabeza et al., 2018, 2019; Montine et al., 2019; Pettigrew and Soldan, 2019; Stern et al., 2019, 2020). In April 2022, the Collaboratory’s consensus framework was posted on the Collaboratory website. Indicating both foresight and openness to different perspectives, the introduction to the Collaboratory framework includes the following statements: “Our aim is to present a well-defined set of operational definitions in order to encourage, advance, and develop research on these concepts. At the same time, we want to encourage investigators who have different views or use a given concept differently to note how their definitions relate or differ with one of those described here. Similarly, this framework provides a basis for describing how the operational definition of another concept differs from those suggested here.”

In keeping with that spirit, here we address what we see as some key issues, and we provide terminology that we believe is clear and can resolve some inconsistencies that are inherent in the Collaboratory’s proposed framework. Many of our terms or concepts are drawn from the extant literature; some are modified, and we also suggest some new related terms. The purpose of this article is not to elucidate mechanisms that may underlie cognitive reserve and related phenomena nor to determine optimal operational definitions, although we provide several reasonable examples. Rather, our goal is simply to provide a consistent set of definitions and constructs that can be applied in efforts to examine cognitive reserve. Despite the substantial body of research on cognitive reserve and related concepts, the field still lacks clear and, most importantly, internally consistent definitions of the basic constructs that can be clearly linked to operational definitions and statistical models. We think that is an important starting point for furthering research and enhancing communication. Conceptual definitions of key terms are shown in Box 1. These are grouped into three major categories: Reserve, Maintenance, and Resilience. Within each category there are parallel definitions for cognition and brain.

Box 1 **Conceptual definitions.**

**Table:** 

**Reserve**	
Cognitive reserve	An individual’s total or overall cognitive resources.
Brain reserve	An individual’s total neural resources or neurobiological capital.
**Maintenance**	
Cognitive maintenance and maintenance of cognitive reserve	The degree to which cognitive decline over time is minimized. Maintenance of cognitive reserve simply refers to maintenance with respect to cognitive reserve specifically (i.e., overall cognitive ability); it highlights the fact that cognitive reserve can change over time.
Brain maintenance	The relative absence of deterioration over time in brain structure or function.
**Resilience**	
Cognitive resilience	The ability to maintain cognitive performance in the face of adverse brain-related change, measured pathology, or other risk factors for cognitive decline.
Brain resilience	Brain structure or function that is better maintained given factors that cause, or increase risk for, adverse brain changes.
Resistance	Avoiding cognitive decline or brain pathology despite adverse factors. Resistance is a subcategory of resilience because resistance against one risk factor necessarily means resilience against some other factor.

*Note: The distinction between maintenance and resilience depends on the presence of a measured or stated risk factor. Lack of decline in the absence of a risk factor is simply maintenance. A risk factor must be identified for there to be resilience or resistance. Thus, resilience or resistance may be viewed as the mechanism allowing for good maintenance despite the presence of a particular risk factor*.

## Inconsistency Between Conceptual and Operational Definitions

In part 1, Definition, in the Collaboratory framework (April 2022)^1^, cognitive reserve is defined as “a property of the brain that allows for cognitive performance that is better than expected given the degree of life-course related brain changes and brain injury or disease.” Inherent in this conceptual definition is the idea that cognitive reserve involves some sort of adaptability to aging-related changes or pathology. We consider this to be a conceptual definition which is followed by part 2, Operational Definition.

There it states that research on cognitive reserve requires three components:

(1) “measures of life course-related changes, insults, or disease or risk factors that theoretically impact cognitive outcomes; (2) measures of associated change in cognition; (3) a variable that influences the relationship between 1 and 2.” An example is provided with “education as a hypothesized cognitive reserve proxy (3)”, which refers to #3 above. Thus, under the Operational Definition subheading, the measure that moderates the relationship between 1 and 2 is the CR (cognitive reserve) proxy.

The following illustrates the logical disconnect, i.e., the incongruence, between the conceptual and operational definitions of cognitive reserve in this framework:

• Cognitive reserve is a property of the brain that allows for cognitive performance that is better than expected given the degree of life-course related brain changes and brain injury or disease.
• Education (or IQ) is a proxy for cognitive reserve.
• Therefore, education (or IQ) refers to a property of the brain that allows for cognitive performance that is better than expected given the degree of life-course related brain changes and brain injury or disease.

Note that the conclusion follows from the statement that education (or IQ) is a proxy for cognitive reserve. A dictionary definition of proxy is “the agency, function, or office of a deputy who acts as a substitute for another (Merriam-Webster Collegiate Dictionary^2^). Thus, we can read this statement to mean that that these are substitutes for cognitive reserve. The problem then is that the last statement about education or IQ is inaccurate. They do not, in and of themselves, indicate or measure anything about a property of the brain nor do they indicate anything about allowing for better-than-expected cognitive performance. Someone with a high IQ or high educational attainment might, for example, perform better than, worse than, or as expected. Therefore, education (or IQ) are not really substitutes for this definition of cognitive reserve. Some readers may feel that what the Collaboratory definition is really intending to say is that education (or IQ) contribute to or are associated with factors that provide the brain with properties that allow for better-than-expected performance. If so, this remains problematic for two reasons. First, it relies on intuition about the meaning of each definition. Second, it confounds the effect with factors that contribute to the effect.

### Moderators vs. Moderation Effects

An interaction showing a significant moderation effect provides the strongest evidence for doing better than expected given aging- or pathology-related brain changes. For example, we might find that memory is correlated with brain pathology in a low, but not a high, education group. The terminology proposed by the Collaboratory creates the logical dilemma in which cognitive reserve (e.g., education) is simultaneously the moderator and the moderation effect itself (e.g., the education × pathology interaction on memory). Put another way, as described above, that approach confounds the effect with factors that contribute to the effect. We can think of no other research paradigm in which it would be acceptable to have the same construct be both moderator and moderation effect. One might argue that this is OK because IQ and education are really measures of adaptability since a person’s total cognitive resources at a given point in time is the sum total of a dynamic process that involves the effects of genes, accumulated experiences throughout life, and adaptability. The same is true for personality traits, body mass index, and myriad other measures that change over the life course. However, despite the fact that each of these measures reflects an endpoint of a set of dynamic processes, the value of that measure only captures a single point in time and it does not necessarily indicate the possibility of better-than-expected performance.

In work that anticipated the Collaboratory, resilience is referred to as “a more general term referring to multiple reserve-related processes” such as cognitive reserve, brain reserve, and brain maintenance (Stern et al., 2020). We note this here because a key part of our resolution of the inconsistency and the blurring of the definitions of moderator and moderation effects involves a specific distinction between reserve and resilience.

## Resolving The Inconsistency: Proposed Definitions

### Reserve

Our proposed resolution is to conceptually define cognitive reserve as a person’s total or overall cognitive resources at a given point in time. This corresponds well to a dictionary definition of reserve as “something set aside for a particular purpose, use, or reason” (Merriam-Webster Collegiate Dictionary^2^). It fits with the notion of reserves as a stockpile of resources. Brain reserve (Satz, 1993) or the similar concept of neuronal reserve (Mortimer et al., 1981) has been used to refer to the amount of premorbid brain tissue. We conceptually define brain reserve as a person’s total neural resources or their neurobiological capital at a given point in time (cf. Arenaza-Urquijo and Vemuri, 2020).

Our definition of cognitive reserve is partly like that of Montine et al. (2019), who define cognitive reserve as a person’s pre-existing or premorbid cognitive ability, i.e., their overall cognitive ability before aging- or disease-related declines. Extending their definition, we also emphasized cognitive reserve as potentially changing over the lifespan. Although we measure it operationally at a particular point in time, reserves may be augmented or depleted across the lifespan. Thus, we differentiate between prior and current level of cognitive reserve. Level and change in cognitive reserve across the lifespan are a function of the interplay of genetic predisposition and environmental influences (Stern et al., 2020). As brain development takes place, cognitive reserve tends to increase primarily early in life through young adulthood, and then tends to become depleted in later life (Zahodne et al., 2015a; Cabeza et al., 2018; Kremen et al., 2019). Importantly, older adults with the same current level of cognitive reserve could still have very different earlier or premorbid levels of cognitive reserve, and *vice versa*. Like cognitive reserve, brain reserve can change across the lifespan. In older adults, it is safe to assume that we are virtually never dealing with prior/peak brain reserve because some amount of atrophy has almost certainly taken place. Thus, we think it is important to acknowledge whether a measure is assessing current or prior reserve as studies may have more or less ability to discriminate between the two.

Given this definition of cognitive reserve, the best available indices, i.e., operational definitions, of cognitive reserve are probably IQ or other measures of general cognitive ability because these essentially measure overall cognitive resources (Boyle et al., 2021). Epidemiological studies have found that higher education is associated with reduced risk for dementia (e.g., Zhang et al., 1990; White et al., 1994; Evans et al., 1997; Beydoun et al., 2014). Subsequently, education has often been used as an operational definition of cognitive reserve. From a neuropsychological perspective, education is commonly used as a rough gauge of premorbid cognitive ability because much more precise actual premorbid cognitive test data are rarely available. Although we believe these are reasonable operational definitions, the choice should ultimately made based on the context of a given study.

In general, more extensive cognitive testing will allow for a better index of general cognitive ability. That said, we are not suggesting that that is required for studying cognitive reserve, and we recognize that extensive cognitive data may not be available, especially for indices of early or premorbid ability. Education or single cognitive measures such as vocabulary or a reading-based premorbid IQ estimate may certainly be employed as cognitive reserve measures. The strengths and limitations of any measures can then be considered. Although we are primarily thinking about cognitive reserve with respect to overall cognitive ability, it is also a valid area of investigation to examine cognitive reserve with respect to a specific cognitive domain. In that case, the index of cognitive reserve would be composed of measures tapping that specific domain as specified by the researchers. Importantly, performing better than expected is not part of our definition of cognitive reserve, although we fully agree that performing better than expected is still of central interest. As described below, our definition of resilience addresses forms of adaptability or performing better than expected.

## Maintenance

The concept of brain maintenance was introduced by Nyberg et al. (2012). It is the relative absence or slower rates of deterioration over time in brain structure or function. Better brain maintenance is simply minimizing deterioration over time, often measured by cortical thinning or volume reductions. As such, this concept does not necessarily apply to childhood and adolescence when brain development, rather than maintenance, is considered optimal. Although weaker inferences may be drawn from cross-sectional findings that are consistent with brain maintenance, longitudinal assessment is necessary to be able to truly determine brain maintenance (see below).

### Cognitive Maintenance and Maintenance of Cognitive Reserve

Here we introduce new terms that parallel Nyberg et al.’s (2012) concept of brain maintenance: cognitive maintenance and maintenance of cognitive reserve. We define cognitive maintenance as the degree to which cognitive decline over time is minimized. We generally use the term cognitive maintenance in regard to measures of specific cognitive abilities. When applied to measures of overall cognitive ability, it may be conceptualized as maintenance of cognitive reserve. In this sense, equivalent prior/premorbid and current cognitive reserve would indicate strong cognitive reserve maintenance. Better cognitive maintenance simply refers to observed lack of decline and can thus be used even when no measures of pathology or other adverse factors are available.

Like brain maintenance, cognitive maintenance (or maintenance of cognitive reserve) really requires longitudinal assessment. In a statistical model, baseline or peak cognitive reserve corresponds to the intercept and cognitive reserve maintenance corresponds to the slope. If two people have high and low prior cognitive reserve, respectively, it may the case that they decline at similar rates, i.e., they have different intercepts but parallel slopes or trajectories. Thus, they would have equivalent cognitive reserve maintenance, but the one who began with a lower intercept will reach the threshold for cognitive impairment or dementia earlier. This scenario has been aptly termed “preserved differentiation” by Salthouse et al. (1990). If the slopes are different and one individual has steeper declines, as tested with an interaction effect, that would indicate a differential cognitive maintenance (i.e., better cognitive maintenance corresponds to a more positive or less negative slope). In the terminology of Salthouse et al. (1990), this scenario has been termed “differential preservation.” Salthouse’s terminology applies equally to maintenance of specific cognitive abilities or maintenance of cognitive reserve.

Using this approach, we previously showed that after controlling for midlife cognitive reserve level, there was still some advantage with respect to specific cognitive abilities in having high cognitive reserve in young adulthood (Eglit et al., 2022). Although declines are usually associated with poorer outcomes, despite being essentially equated for current cognitive reserve, this high declining group performed better on specific cognitive abilities in mid- and later life. We referred to this as a paradoxical reserve phenomenon because of the advantages shown in the declining group.

We suspect that a major reason for the lack of reference to maintenance of cognitive reserve in the literature is due to education and reading-based premorbid IQ estimates being the most common indices of cognitive reserve. In studies of older adults, it is not possible to examine change in reserve based on these indices.

## Resilience

Resilience is a generic term that could be applied to cognition, brain, depression and many other constructs. In keeping with our effort to define parallel terms for cognition and brain, we think it is important to specify cognitive or brain resilience. We define cognitive resilience as the ability to maintain cognitive performance in the face of adverse brain-related change, measured pathology, or other risk factors for cognitive decline. Like our definition of cognitive reserve, this corresponds to a dictionary definition of resilience as “the ability to recover from or adjust easily to misfortune or change” (Merriam-Webster Collegiate Dictionary^2^).

Inferring cognitive resilience requires some adverse change or factor that a person can be resilient against. Thus, cognitive maintenance in the absence of any known or specified adverse factor would not come under the rubric of resilience. Cognitive performance might, for example, be resilient against Alzheimer’s disease pathology (amyloid and tau) or neurodegeneration. Carrying the *APOE*-ε4 allele or experiencing environmental exposures such as air pollution, which can also have adverse brain effects, are examples of additional factors one may be resilient against. In a statistical model, these types of cognitive resilience would be operationally defined as interaction effects (e.g., that young adult cognitive reserve moderates the association between pathology and current cognitive performance, or that among *APOE*-ε4 carriers, young adult cognitive reserve moderates the association between medial temporal lobe volume and episodic memory).

**Figure 1:**
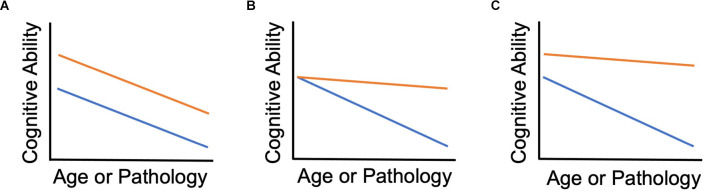
Cognitive reserve and cognitive resilience. **(A)** Orange group has higher reserve but no difference in resilience. **(B)** Baseline reserve is equal between groups; Orange group shows resilience over time compared to the blue group. **(C)** Orange group has both higher baseline reserve and shows resilience over time.

Figure 1 depicts the relationship between cognitive reserve and resilience in our framework. The figure provides three examples of longitudinal scenarios. Our proposed terminology is applicable to cross-sectional studies as well, but we recommend that findings from cross-sectional data are framed as “consistent with” resilience rather than definitive evidence. Baseline in each panel refers to the earliest point at which cognitive reserve is measured in a given study. Each panel is consistent with normative decline, i.e., the most common scenario of depletion of cognitive reserve with advanced aging (cf. Zahodne et al., 2015a). Figure 1A depicts an advantage of higher cognitive reserve that is present at baseline and remains constant over time with no evidence of resilience. This is what Salthouse referred to as preserved differentiation. Figure 1B depicts a situation in which two groups have equivalent baseline cognitive reserve. Assuming some pathological factor, the orange group exhibits greater cognitive resilience because they have less cognitive decline (alternatively, better maintenance of cognitive reserve). Figure 1C depicts a case in which one group has both greater baseline cognitive reserve and greater cognitive resilience (alternatively, better maintenance of cognitive reserve). In Salthouse’s terminology, Figure 1B would be an example of differential preservation and Figure 1C could arguably show both differential preservation and preserved differentiation. Because the less steep slope in Figures 1B,C implies performance that is better than expected, it corresponds to the final April 2022 Collaboratory framework definition of cognitive reserve. In contrast to our usage, resilience is a general, overarching term in the Collaboratory framework (Stern et al., 2020), so there is no real differentiation between reserve and resilience. But with that definition of cognitive reserve, it no longer seems relevant for later life interventions to improve cognitive function because it is important to differentiate whether the effects of interventions are on reserve or resilience. In this case, the orange lines would represent the group undergoing the intervention. Individuals participating in cognitive training and their families are most likely to want to know whether it will boost their cognitive resilience (i.e., slower rate of decline compared to no intervention) rather than simply boost cognitive reserve (i.e., baseline post-intervention functioning higher than pre-intervention) with no impact on rate of decline.

Directly paralleling our conceptualization of cognitive resilience, we define brain resilience as brain structure or function/activation that is better maintained given factors that cause, or increase risk for, adverse brain changes. Evidence of brain resilience thus requires evidence of these deleterious factors. Examples of brain resilience might be evidence of higher-than-expected hippocampal volume given *APOE*-ε4 homozygosity or pathological levels of beta-amyloid.

Because cognition arises from brain, cognitive resilience must ultimately be due to brain resilience. Thus, it does not seem possible for the two to be entirely independent, i.e., to have cognitive resilience without brain resilience. On the other hand, different scenarios are conceivable. If we examine amyloid accumulation as a type of brain pathology, then brain resilience might be manifest by reduced hippocampal atrophy over time. Trajectories of episodic memory decline that are less steep than trajectories of hippocampal atrophy would additionally constitute cognitive resilience. However, if the trajectory of episodic memory decline always parallels that of hippocampal atrophy, that would not represent cognitive resilience. In the latter case, cognitive performance is not better than expected given the level of brain resilience.

Arenaza-Urquijo et al. (2017) and Arenaza-Urquijo and Vemuri (2020) have proposed that aging and dementia research will benefit from a distinction between resilience and resistance. Like their definition, we define resistance as avoiding pathology or cognitive decline despite the existence of some adverse factor. A risk factor must be identified for there to be resilience or resistance. In the absence of a risk factor (or if a risk factor is not specified), manifesting good performance or the lack of decline or pathology is simply maintenance. We consider resistance to be a subtype of resilience because it is not possible to be resistant without being resilient against something else. Which term is applicable may simply depend on whether one is looking “upstream” or “downstream.” If someone has low amyloid levels despite being *APOE*-ε4 homozygous, then they are resistant to amyloid accumulation but they are—by definition—also resilient against their high genetic risk.

## Implementation in Research

Two examples illustrate how our proposed usage can be applied in empirical research and how it avoids conflating moderator and moderation effects. We previously showed that the association between hippocampal volume and episodic memory was moderated by young adult general cognitive ability, i.e., young adult cognitive reserve (Vuoksimaa et al., 2013). Memory performance and hippocampal volume were positively correlated in the lower reserve group, but not in the higher reserve group. The results are consistent with the idea that higher cognitive reserve is protective against the effects of hippocampal atrophy, so that memory in those with high reserve was less dependent on hippocampal volume. In 2013, we referred to both young adult general cognitive ability and the moderation effect as cognitive reserve. In our current proposed usage, we would say that higher cognitive reserve appeared to confer greater cognitive resilience in the face of smaller hippocampal volume. In another study, we showed that the same young adult measure of cognitive reserve moderated the association between midlife lifestyle and later brain aging (Franz et al., 2021). Favorable lifestyles were associated with less advanced brain aging among individuals with lower young adult cognitive reserve, but individuals with higher young adult cognitive reserve had less advanced brain aging regardless of lifestyle. In this case, we concluded that higher young adult cognitive reserve conferred cognitive resilience against the adverse effects an unfavorable lifestyle on brain aging. In addition, we concluded that a favorable lifestyle conferred resistance to advanced brain aging because it was associated with little advanced brain aging. This result might also reflect brain maintenance, but we were reluctant to make that inference because brain age was measured at only a single time point.

### Parallel Concepts for Cognition and Brain

Some researchers argue that we should use a single term for reserve without differentiating between cognitive and brain reserve because all cognition arises from the brain (Cabeza et al., 2018). Others, however, argue that it is important to distinguish between the two (Stern et al., 2020). Although the former raises a valid conceptual point, we agree with the latter position for practical reasons. It is, of course, true that cognition arises from the brain, but people with very similar brain pathology may have vastly different levels of cognitive function, and those with similar cognitive function may have very different brains. Indeed, studies reviewed by Cabeza and colleagues indicate that the same cognitive performance has been associated with different patterns of brain activity in younger and older adults (Dolcos et al., 2002). In our view, this lack of one-to-one correspondence between brain and cognition suggests that it is useful to retain separate terms for cognitive reserve and brain reserve. With continued advancement in mapping the brain, that correspondence may become closer. As a result, the importance of brain reserve might increase relative to that of cognitive reserve.

Some researchers may suggest that brain structure captures brain reserve whereas brain function/activation captures cognitive reserve. A brain activation pattern (e.g., as observed in fMRI) that is linked to overall cognitive ability may serve to elucidate mechanisms underlying cognitive reserve, but in our view, it would not be considered to operationally define cognitive reserve because the functional brain activation pattern itself is not cognition. Similarly, we can assume that some particular pattern of brain activation involving motor, memory, and association areas underlies performance of a pirouette in ballet, but we would not want to define that brain activation pattern as a pirouette.

### Issues Regarding Cognitive Reserve Proxies

In commentaries on reserve (Cabeza et al., 2018; Stern et al., 2020), it is stated that reserve is a construct or concept that cannot be measured directly and can only be measured *via* proxies. Personality is assessed with a variety of personality inventories. Memory may be assessed by story or list recall. Personality, memory, intelligence, and myriad others are theoretical or latent constructs that cannot be measured directly. In our view, referring to operational definitions of cognitive reserve as proxies tends to convey that cognitive reserve is somehow different—more cryptic and more difficult to measure—than other behavioral or psychological constructs. If the proxy is actually a substitute for the construct (as number of words recalled from a list is a substitute for the construct of episodic memory), then use of the term would not be problematic. Of course, it could then simply be referred to as the operational definition in line with conventional scientific usage. However, as explained through our syllogism example, the so-called proxies do not constitute substitutes for cognitive reserve as defined by the Collaboratory. The term proxy does not get around the inconsistencies we have noted between operational and conceptual definitions of reserve. This holds true whether the so-called proxy is a cognitive measure (e.g., vocabulary) or a lifestyle measure (e.g., engagement in cognitive activities). The importance of considering how an operational measure is related to conceptual definitions is discussed in the next section.

### Differentiating Cognitive Reserve From Factors That May Influence Cognitive Reserve or Resilience

We are concerned regarding what we see as dilution of the concept of cognitive reserve. Cognitive reserve is determined by genetic factors plus accumulated experiential factors that may tend to enhance or reduce the level of reserve. Experiential/environmental factors might, for example, include nutrition, physical activity, exposure to education, exposure to air pollution, occupational complexity, and engagement in cognitively stimulating activities. These measures—individually or in composite indices—have often been used as so-called proxies for cognitive reserve. Even socioeconomic status has sometimes been included in measures of cognitive reserve. We strongly argue against this practice. These measures may contribute to one’s cognitive reserve (i.e., their overall cognitive capacity), or cognitive resilience (i.e., better-than-expected performance), or they could be the product of cognitive reserve. But they are not measures of cognitive resources or capacity, nor do they say anything in and of themselves about better-than-expected performance. Low physical activity, for example, may contribute to obesity, but it makes no sense to suggest it as a proxy (substitute) for obesity. These other measures may be included in data analytic models, as variables that may affect cognitive reserve or be partially driven by cognitive reserve rather than as indices of cognitive reserve themselves. The way in which a given measure may be related to reserve should simply be described (e.g., “measure X contributes to reserve” or “measure X is the product of high reserve”). This distinction is also important because factors that contribute to cognitive reserve may represent potential targets for intervention. Finally, including socioeconomic status as part of a cognitive reserve measure is particularly problematic given the potential implication that simply being poor means one has low cognitive reserve.

We make an exception for education as an indicator of cognitive reserve—even though it is not a direct measure of cognitive ability—because people with greater cognitive capacity tend to attain higher levels of education. Because early cognitive data are rarely available, it is traditional in neuropsychological assessment to use education as a rough way to gauge a person’s expected level of cognitive function. Consequently, education is often used to estimate peak cognitive reserve. That said, it is worth noting that education is a rather crude index of cognitive reserve. With that in mind, we showed that a measure of general cognitive ability taken during young adulthood, but not education, moderated the correlation between memory and hippocampal volume (Vuoksimaa et al., 2013). A similar finding has subsequently been shown for current verbal intelligence compared with education (Boyle et al., 2021).

### Cognitive Reserve and the Question of Reverse Causation

A key drawback of using variables other than direct cognitive measures—including education—as indicators of cognitive reserve is the “chicken-egg” problem. We referred to factors that *may* influence reserve in order to avoid assumptions about direction of effect. For example, does engaging in cognitively stimulating activities reduce age-related declines or is it that individuals with higher cognitive reserve tend to engage more in cognitive-related activities? Does getting more education increase one’s cognitive reserve or do people with more cognitive reserve tend to attain higher levels of education? There is evidence that during childhood and adolescence—when there is substantial brain development—that education provides an environment that promotes increases in IQ or general cognitive ability (Ritchie and Tucker-Drob, 2018). However, that effect appears to level off by young adulthood (Kremen et al., 2019). We have shown, for example, that after accounting for a direct measure of general cognitive ability administered in young adulthood, education, occupational complexity, and engaging in cognitive activities later in life accounted for less than 1% of the variance in later cognitive function (Kremen et al., 2019). These results are consistent with reverse causation, i.e., that the impact of education, occupational complexity, and cognitive activities *after early adulthood* was not truly causal.

The possibility of reverse causation receives far too little attention in studies of cognitive reserve. This possibility is another reason to avoid considering measures such as engagement in leisure activities or occupational complexity to be indices of cognitive reserve. For example, engaging in leisure activities might enhance cognitive reserve, which in turn may enhance cognitive resilience. However, higher cognitive reserve may make someone more inclined to engage in leisure activities, so leisure activities may be associated with cognitive resilience due to reverse causation. Alternatively, both could independently be associated with cognitive resilience. Using leisure activities as a proxy or measure of cognitive reserve or including it in a composite reserve measure precludes examining these possibilities.

To be able to infer causal effects of educational or cognitively stimulating exposures later in life and avoid the problem of reverse causation, individuals must be randomly assigned to conditions. With random assignment, cognitive training programs of various kinds have shown some modest improvements in cognitive function and/or brain structure (Willis et al., 2006; Hertzog et al., 2008; Park et al., 2014; Carlson et al., 2015; McDonough et al., 2015; Motes et al., 2017). Consistent with our conclusion that young adult cognitive ability is the primary driver, the successful training programs have tended to involve intensive and lengthy training with relatively small effects. However, if those effects help to slow decline, they may be well worth it.

### Considerations for the Use of Residual Scores

In some cases, researchers may wish to examine residual scores to determine if cognitive function is better or worse than expected given some indices of their brain status or some adverse risk factor. Studies of cognitive reserve and resilience using this approach have been subjected to a meta-analysis by Bocancea et al. (2021). An example of this approach would be regressing memory performance on level of beta-amyloid. A positive residual score indicates that the observed memory score is higher than the score predicted by the regression equation. A negative residual indicates lower than expected performance. Although this may be labeled as higher and lower cognitive reserve, we note that under our terminology it would more closely reflect cognitive resilience. However, if the measures do not necessarily indicate adverse factors (e.g., cross-sectionally measured hippocampal volume without evidence of atrophy), then it does not provide strong evidence of resilience.

Critically, the use of residual scores to assess resilience is dependent on the association between the predictor and outcome variables (Elman et al., 2022). For this approach to be meaningful, the magnitude of the correlation between the relevant variables (e.g., brain structure and memory) must be relatively high. If these measures are only modestly correlated, as is often the case when correlating cognitive scores with MRI measures of brain structure or measures of pathology, then the residual memory score will be very highly correlated with original memory score. As such, the residual score may provide little or no predictive value that is not already provided by the original memory score. This is true for change in residual scores over time vs. change in original scores over time.

Longitudinally, one could also compare observed vs. predicted memory scores at time 2 given one’s memory at time 1. One could then examine whether lesser or greater than expected decline based on residual scores was predictive of future outcomes. However, because those with greater than expected decline will also tend to have lower scores at time 2, it is important to test whether the residual score is significant over and above the person’s score at time 2 (Kremen et al., 1998; Franz et al., 2019). Essentially, this addresses the question of which is the primary determinant: how the person got there (i.e., their trajectory) or where they are at currently?

### What Constitutes High or Low Cognitive Reserve?

Within a given study of cognitive reserve, it is common to classify individuals as low or high reserve. Those classifications are typically made relative to others within a particular study sample and may have little comparability across different studies. What constitutes low reserve may be particularly important for Alzheimer’s-related studies. Several major studies of Alzheimer’s disease comprise older adults whose average education is very high relative to their cohort in the population. Consider, for example, a study that began in 2005 with an average baseline age of 72 and average education of approximately 16 years. The average birth year would be 1933. U.S. census data show how highly atypical this educational level is in that only about 10% of that cohort attained a 4-year college degree (Ryan and Bauman, 2016). This issue has significant implications for the study of Alzheimer’s disease given that low education—the most commonly used index of cognitive reserve—is associated with increased risk for dementia.

The relevance of defining low and high cognitive reserve is illustrated with data from four studies in which participants were divided into high and low reserve groups. In the study of Yaffe et al. (2011), there were two indices of cognitive reserve. One index defined low reserve (55% of the sample) as less than a high school education. The other index defined low reserve (15% of the sample) as literacy at or below the sixth-grade level. The sample of Soldan et al. (2017) had a mean education level of 17.0 (SD = 2.4; Soldan et al., 2017). The high and low cognitive reserve group in the sample of Vuoksimaa et al. (2013) had 14.9 (SD = 2.6) and 12.9 (SD = 1.9) years, respectively. Zahodne et al. (2015b) had a high education group with 9–20 years and a low education group with 0–8 years of education (Zahodne et al., 2015b). What would be the high reserve group in the studies of Yaffe et al. or Zahodne et al. would cover both groups in the study of Vuoksimaa et al. Being two SDs below the mean in the study of Soldan et al. (2017) would correspond to about the middle of the low reserve group in the study of Vuoksimaa et al. (2013) and would place someone solidly within what would be the high reserve group in the studies of Yaffe et al. and Zahodne et al. These were well-designed studies, but they highlight the fact that the meaning of high and low reserve may be radically different across studies. As such, the inferences we make about such comparisons may not translate well from one study to another. More attention should thus be paid to this issue when comparing results across studies. Because indices of cognitive reserve often do not have a standardized scale, the correspondence of high and low reserve across studies should be addressed when comparing results across studies.

## Summary and Conclusions

Cognitive reserve and related constructs have proven to be valuable tools for aging-related research, but we believe greater consistency in the definition of terms both within and between studies is still needed to improve communication. Greater clarification in the definition of terms should also improve study design and inferences made on the basis of study results. Key points regarding these concepts and definitions are as follows: (1) cognitive reserve can be defined as overall cognitive resources *at a given point in time*; (2) as such, we suggest that prior/peak vs. current cognitive reserve be specified; (3) like brain maintenance, cognitive (reserve) maintenance is a dynamic process that must be evaluated longitudinally; (4) cognitive resilience is evidence of a dynamic process that involves maintaining performance in the face of pathology or some other adverse factor; (5) resistance can be viewed as a subcategory of resilience because resistance against some downstream adverse factor necessarily implies resilience against some upstream factor; (6) given our current knowledge and measurement ability, it is useful for researchers to have parallel terms for each of these applying to brain and cognition; (7) it is important to clearly differentiate between reserve and factors that may influence reserve; (8) greater attention to the possibility of reverse causation is warranted; (9) greater caution is warranted in making causal inferences about these phenomena in cross-sectional studies; (10) attention to the definitions of low and high cognitive reserve within studies will be important to enhance our ability to compare results across studies.

We believe our terminology lends itself to a clear analytic framework, eliminates confusion, avoids logical inconsistency between conceptual and operational definitions, and clearly differentiates between moderator and moderation effects. The next steps will be to identify the specific underlying process or mechanism that accounts for higher cognitive reserve conferring greater resilience.

## Author Contributions

WK, JE, MP, GE, and CF contributed to the conception and design of the manuscript. WK wrote the first draft of the manuscript. WK, JE, MP, GE, and CF wrote sections of the manuscript. All authors contributed to the article and approved the submitted version.

## Conflict of Interest

The authors declare that the research was conducted in the absence of any commercial or financial relationships that could be construed as a potential conflict of interest.

## Publisher’s Note

All claims expressed in this article are solely those of the authors and do not necessarily represent those of their affiliated organizations, or those of the publisher, the editors and the reviewers. Any product that may be evaluated in this article, or claim that may be made by its manufacturer, is not guaranteed or endorsed by the publisher.

## Footnotes

^1^ https://reserveandresilience.com

^2^ http://www.merriam-webster.com/dictionary
